# Encapsulated LyeTx III Peptide: Cytotoxic Agent Isolated from *Lycosa erythrognatha* Spider Venom

**DOI:** 10.3390/toxins17010032

**Published:** 2025-01-10

**Authors:** Daniel Moreira dos Santos, Livia Ramos Santiago, Nayara Araújo dos Santos, Wanderson Romão, Jarbas Magalhães Resende, Maria Elena de Lima, Márcia Helena Borges, Rosy Iara Maciel de Azambuja Ribeiro

**Affiliations:** 1 Department of Experimental Pathology, Federal University of São João del-Rei, Divinópolis 36301-158, Brazil; santosdm@gmail.com (D.M.d.S.); santiagoliviar@gmail.com (L.R.S.); 2 Petroleomics and Forensics Laboratory, Federal University of Espírito Santo, Vitória 29075-910, Brazil; nayara.ads@gmail.com (N.A.d.S.); wandersonromao@gmail.com (W.R.); 3 Department of Chemistry, Federal University of Minas Gerais (UFMG), Belo Horizonte 30110-005, Brazil; jmr@ufmg.br; 4 Programa de Pós-Graduação em Medicina-Biomedicina, Faculdade Santa Casa de Belo Horizonte, Belo Horizonte 30110-005, Brazil; 5 Proteomics and Arachnid Laboratory, Ezequiel Dias Foundation, Belo Horizonte 30110-005, Brazil; mhborgesb@gmail.com

**Keywords:** antimicrobial peptides, antitumor activity, spider venom, encapsulated peptide

## Abstract

The discovery of novel cytotoxic drugs is of paramount importance in contemporary medical research, particularly in the search for treatments with fewer side effects and higher specificity. Antimicrobial peptides are an interesting class of molecules for this endeavor. In this context, the LyeTx III, a new peptide extracted from the venom of the *Lycosa erythrognatha* spider, stands out. The peptide exhibits typical antimicrobial traits: a positive net charge and amphipathic α -helix structure in lipid-like environments. Its unique sequence (GKAMKAIAKFLGR-NH_2_), identified via mass spectrometry and Edman degradation, shows limited similarity to existing peptides. Significantly, when liposome-encapsulated, LyeTx III demonstrates selective activity against tumor cells in culture. Our MTT results showed that the cytotoxicity of the peptide increased against HN13 cells when administered as liposomes, with their viability in HN13 cells alone being 98%, compared to 38% in liposome-encapsulated form. This finding underscores that the LyeTx III peptide may be a good candidate for the development of new drugs against cancer. Its activity when encapsulated is promising, as it can increase its half-life in the body and can also be targeted to specific tumors.

## 1. Introduction

Antimicrobial peptides (AMPs) are a diverse class of molecules that possess, in addition to antimicrobial activity, immunomodulatory and anticancer activities [1]. In recent years, there has been increasing interest in exploring the potential of spider-venom-derived AMPs as anticancer agents due to their potent cytotoxicity against tumor cells [2].

Several studies have reported the ability of animal-venom-derived AMPs to selectively target and induce apoptosis or other mechanisms of death in diverse types of tumor cells, including breast [3,4,5], lung [6], prostate [7], glioblastoma [8], and colon [9] cancer cells. The mechanisms underlying the cytotoxic effects of these peptides on tumor cells include the disruption of the plasma membrane; induction of oxidative stress; and activation of apoptotic, autophagic, necroptosis, or other pathways [3,4,8,10]. For example, Cathelicidin LL-37 induces apoptosis in colon cancer cells via caspase-3 activation and mitochondrial disruption [11,12], while Buforin IIb, derived from histone H2A, penetrates tumor cells without membrane damage, leading to mitochondria-dependent apoptosis [13]. Temporin-1CEa, from Chinese brown frog, disrupts the tumor membrane [14], whereas human β-defensin 3 (hBD3) binds to phosphatidylinositol 4,5-bisphosphate on cell membranes, causing cytolysis. Temporin-1a, from bullfrog skin, acts by disrupting the tumor membrane, while human β-defensin 3 (hBD3) binds to phosphatidylinositol 4,5-bisphosphate [PI(4,5)P2] on the cell membrane, leading to cytolysis. Finally, the synthetic peptide KI-21-3, a fragment of LL-37, has also demonstrated antitumor effects in oral squamous cell carcinoma. The research on these and other antitumor peptides continues to progress, seeking new therapies for cancer.

Notably, spider-venom-derived AMPs exhibit several advantages over conventional chemotherapeutic agents, including their selectivity towards cancer cells and low toxicity towards normal cells [15]. Additionally, some spider-venom-derived AMPs have been shown to exhibit synergistic effects when combined with other anticancer agents, further highlighting their potential as a new generation of cancer therapeutics cells [4,16].

Our group showed that the antimicrobial peptide LyeTx1-b, a modified peptide from LyeTxI that is purified from the venom of the spider *Lycosa erythrognatha* [17], was cytotoxic to the glioblastoma lineage U87-MG and only mildly cytotoxic against normal fibroblasts of human and monkey cell lines and low-level hemolytic in human erythrocytes [8]. In addition, the systemic administration of this peptide in healthy BALB/C mice did not alter the hematological parameters, except for a reduction in platelet counts [3]. The anticancer property of this peptide was associated with a membranolytic effect being its main mechanisms, suggested to be necroptosis and autophagia in glioblastoma and triple-negative breast cancer cells, respectively.

In this work, using the techniques of high-performance liquid chromatography, mass spectrometry, and automatic Edman degradation, we identified a new peptide from the venom of the spider *Lycosa erythrognatha,* named LyeTx III, with structural characteristics of antimicrobial peptides. Its functional characterization showed lytic activity on liposomes and selective action on the HN13 cell line. Its activity, combined with its small size and ease of synthesis, makes this peptide a good candidate for development as an antitumor drug.

## 2. Results

### 2.1. Purification, Sequencing, and Synthesis of the Peptide

**Figure 1:**
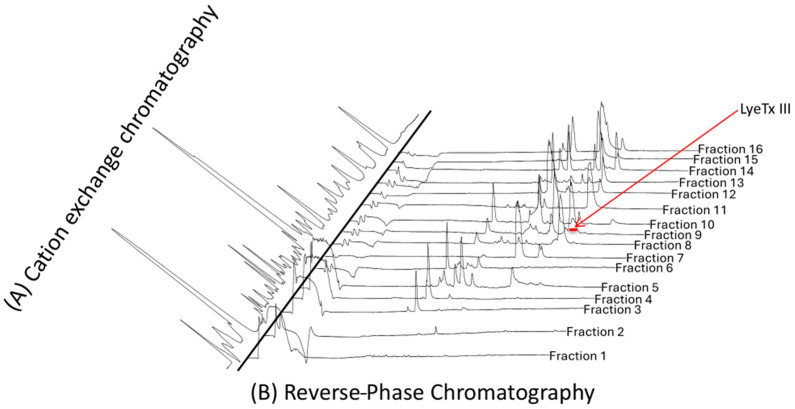
Purification of LyeTx III from spider venom by HPLC. (**A**). The supernatant of the centrifuged crude venom was applied to cation exchange HPLC (TSK gel CM-SW column, 4.6 mm × 250 mm, Tosoh), with a linear NaCl gradient up to 1 M. The flow rate was 0.75 mL·min^−1^, and detection was carried out at 214 nm. (**B**). The fractions were re-purified by reverse-phase HPLC in a C18 Supercosil column (4.6 mm × 250 mm, Supelco) that was equilibrated with 0.1% aqueous trifluoroacetic acid (TFA), followed by a linear gradient of acetonitrile in 0.1% TFA. The flow was 0.75 mL·min^−1^, and detection was carried out at 214 nm. The arrow shows the fraction of peptide LyeTx III.

The crude venom of the spider *L. erythrognatha* was fractionated by CIEX in high-performance liquid chromatography (HPLC), as described in the Methods section, and 16 fractions were manually collected and vacuum-dried, as shown in Figure 1A. All fractions were resuspended in distilled water and purified by RP chromatography, and the major fractions were collected (Figure 1B).

All collected fractions were analyzed by AutoFlex III MALDI-ToF mass spectrometry. We identified 102 unique *m*/*z*. 23 peptides showing *m*/*z* compatibility with more common AMPs (1500–3000 Da).

**Figure 2:**
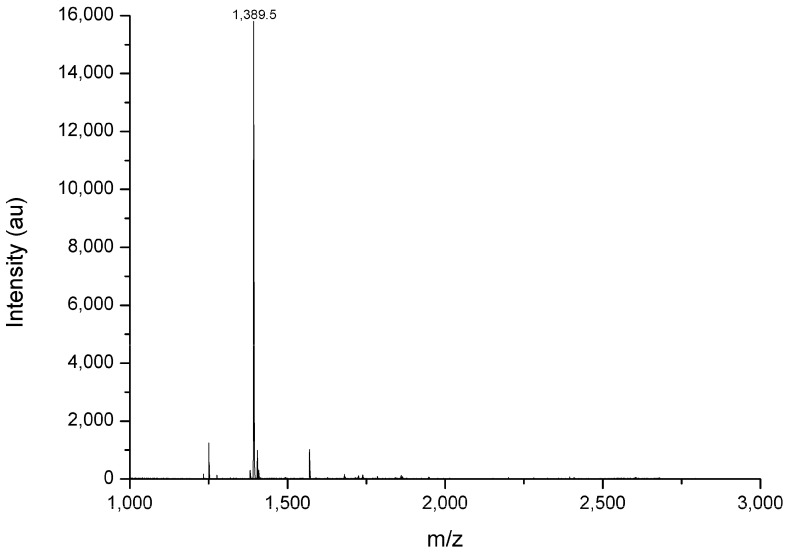
*m*/*z* determined by MALDI-ToF (Autoflex III–Bruker Daltonics) in positive linear mode.

The target peptide was eluted using 0.41 M NaCl in cation exchange chromatography and 46% acetonitrile in reverse-phase chromatography. The reverse-phase chromatography (RPC) containing the peptide showed contamination with two other peptides with *m*/*z* of 1253 and 1570. Despite this contamination, it was possible to determine its primary structure using automated Edman degradation and Matrix-Assisted Laser Desorption Ionization–Time of Flight mass spectrometry (MALDI-ToF) and Fourier transform ion cyclotron resonance mass spectrometry (FT-ICR MS) (Figure 2). The peptide LyeTx III showed a *m*/*z* 1390.75, with a C-terminus amide. Its sequence had 13 amino acids (Table 1) and showed low similarity with those of other AMPs deposited in databases.

To study the physicochemical and bioactive properties of the LyeTx III peptide, it was synthesized using the Fmoc solid-phase strategy. The purification of the peptide was carried out by reverse-phase chromatography in an HPLC system, and the mass and sequence were verified by MALDI-ToF mass spectrometry.

### 2.2. Permeabilization of Liposomes

**Figure 3:**
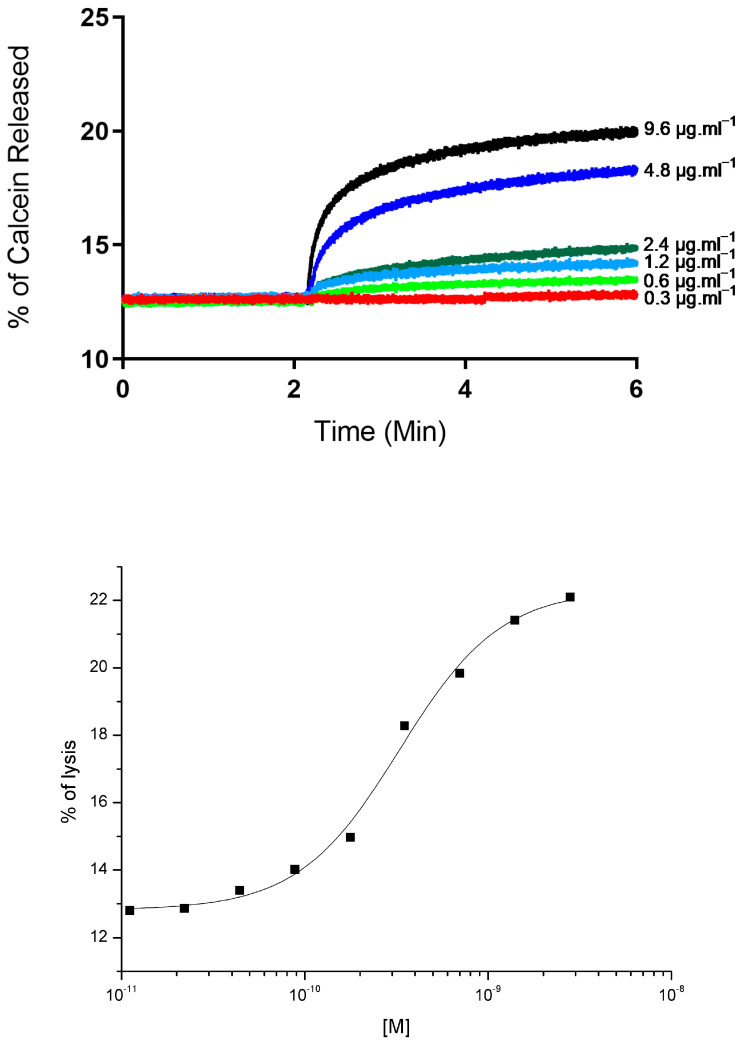
Calcein release from liposomes provoked by LyeTx III. (**A**) Rate of calcein leakage was monitored in liposomes composed of L-α-phosphatidylcholine. LyeTx III was introduced to liposome mixture at varying concentrations, two minutes after incubation at 37 °C. (**B**) Dose–response curve of calcein release induced by LyeTx III in POPC. Fluorescence emission readings were taken four minutes following exposure to toxin.

Upon the addition of LyeTx III (0.6 µg·mL^−1^) to L-α-phosphatidylcholine liposomes (POPC), the entrapped self-quenched calcein was immediately released, as it became evident following the increase in fluorescence intensity (Figure 3A). The maximum release of calcein was approximately 22%. The increments of fluorescence 2 min after adding the peptide are shown in Figure 3B.

### 2.3. Circular Dichroism Spectroscopy

**Figure 4:**
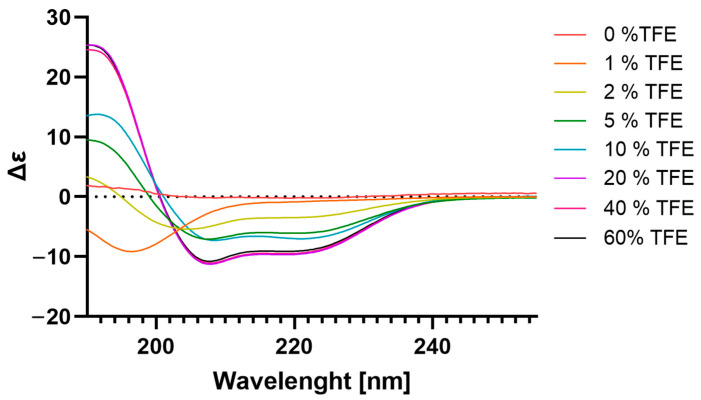
Secondary-structure CD spectra of LyeTxIII at different TFE percentages. The experiment was carried out by incubating the peptide at a concentration of 0.1 mg·mL^−1^ in 0.02 mM (0.6%) TFE to 20 mM (60%) TFE, scanning the spectrum from 190 to 280 nm and using 4 accumulations in a spectropolarimeter Jasco-715 (Jasco, Tokyo, Japan).

**Figure 5:**
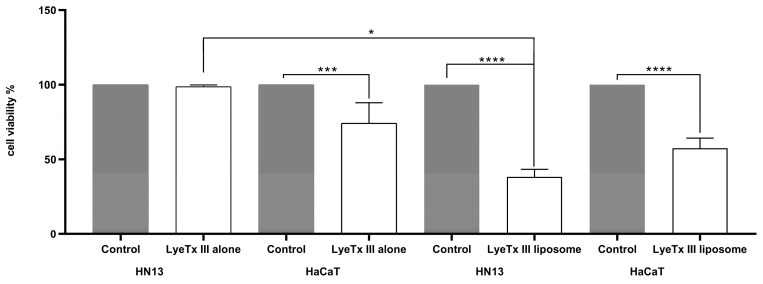
LyeTx III liposome reduced HN13 cells’ viability. LyeTxIII peptide alone at 45 µM and LyeTxIII peptide at 45 µM, encapsulated in liposome. *, ***, and ****: statistical significance level *p* < 0.05, *p* < 0.0005, and *p* < 0.0001, respectively.

As shown in Figure 4, whereas the peptide exhibited predominantly random coil conformations in 2,2,2-trifluoroethanol (TFE) at lower concentrations (<2%), characteristic curves of α-helix formation were obtained upon the addition of 5% TFE (Figure 5). This could be verified by the increase in the CD amplitudes of the characteristic α-helix signals, with minima at 208 and 222 nm and a maximum at 192 nm.

### 2.4. Decreased Cell Viability After Liposome Treatment with LyeTx III Peptide

The cell viability results showed that treatment with LyeTx III 45 μM did not lead to any statistical difference for the tumor cell line, demonstrating that it was more toxic for the non-tumor cell line (Table 2). Figure 5 shows that the encapsulated peptide reduced the cell viability by 62% for the tumor and 46% for the non-tumor cell lines.

## 3. Discussion

The discovery of new cytotoxic drugs, particularly from novel sources like the spider *Lycosa erythrognatha*, is of great importance in the ongoing battle against cancer. Current treatment options often face challenges such as drug resistance, limited efficacy against diverse cancer types, and significant side effects [20]. The exploration of bioactive compounds, especially peptides, presents a promising alternative due to their specificity and reduced toxicity. Peptides can target unique cancer cell pathways, offering the potential for more effective and personalized treatments [21]. Moreover, the natural diversity of organisms like spiders serves as a rich reservoir for novel drug discovery, underscoring the need for continued research in this area [22].

The development of peptides as models for cytotoxic drugs can represent a significant advance in cancer therapy due to their high specificity and reduced toxicity compared to traditional therapies [23]. The shift from conventional treatments to molecular targeting methods highlights the role of anticancer peptides in targeted therapy, offering precise attacks on cancer cells with minimal side effects [24].

The selectivity of antimicrobial peptides (AMPs) for tumor cells over normal cells is primarily attributed to distinct differences in the cell membrane compositions of these two cell types. Tumor cells generally exhibit a higher density of negatively charged molecules, such as phosphatidylserine [25] and specific glycoproteins [26], on their outer membrane. These negatively charged components enhance the electrostatic attraction of positively charged AMPs, facilitating their preferential binding to cancer cell membranes [27]. In contrast, normal cells maintain a more neutral surface charge, reducing the AMP affinity and thereby minimizing the peptides’ cytotoxic effects on healthy tissue.

Using ion exchange and reverse-phase chromatography, followed by sequencing by Edman automatic degradation and fragmentation by mass spectrometry, we obtained the sequence of the LyeTx III peptide (GKAMKAIAKFLGR-NH_2_). Its sequence presents amphipathicity and a positive net charge, important characteristics in AMPs. A positive net charge enables these peptides to interact with the negatively charged cell membranes of microbes and tumor cells, facilitating disruption or penetration [28]. Amphipathicity, the presence of both hydrophilic and hydrophobic parts, allows AMPs to insert themselves into lipid bilayers, which is crucial for their membrane–lytic activity [29]. Also, their small size contributes to their ability to penetrate tissues and reach target cells effectively. Our circular dichroism analysis indicated that the peptide presents an α-helix structure under conditions that simulate a lipid membrane, another characteristic that has been observed in AMPs. This feature is important for directing the hydrophobic and hydrophilic regions during interaction and insertion into the plasma membrane [30].

The sequence showed low similarity with other antimicrobial peptides that are found in the *Lycosidae* family (Table 1). Unique peptide sequences can target specific microbial pathogens or cancer cells with novel mechanisms of action. Additionally, distinct peptide structures may offer new ways to bypass the limitations of current treatments, addressing issues like toxicity and specificity. The development of new antimicrobial or antitumor peptides with sequences that have low similarity with peptides in the sequence database is crucial for broadening the spectrum of therapeutic agents. Unique peptide sequences can target specific microbial pathogens or cancer cells with novel mechanisms of action, potentially reducing the likelihood of resistance development [31]. Additionally, distinct peptide structures may offer new ways to bypass the limitations of current treatments, addressing issues like toxicity and specificity [32].

The LyeTx III peptide was tested on liposomes composed of POPC, where it demonstrated activity. The peptide caused liposome leakage of calcein starting from a dose of 0.6 µg·mL^−1^. Notably, the dose–response curve for LyeTx III’s activity on these liposomes exhibited a sigmoid shape. This particular shape of the curve indicates a cooperative action in the lysis of the membrane [33]. The maximum lysis observed for LyeTxIII was 22.5% at the highest concentration tested and showed an ED50 of 3.28 × 10^−10^ M. This result is significantly lower compared to the LyeTx I peptide, which presents an ED50 of 2.7 × 10^−10^ M for 95% lysis [17]. These data demonstrate the feasibility of using liposomes as LyeTx III peptide carriers.

The encapsulation of peptides, including AMPs, plays a critical role in facilitating their passage across the blood–brain barrier (BBB), a highly selective semipermeable border that separates the circulating blood from the brain and extracellular fluid in the central nervous system [16]. The BBB’s primary function is to protect the brain from harmful substances, but this also makes it challenging for therapeutic agents, such as AMPs, to reach the brain.

Peptides are typically of a high molecular weight and are hydrophilic, properties that limit their ability to penetrate the BBB. The encapsulation of these peptides in specially designed carriers, such as liposomes, polymeric nanoparticles, or nanogels, can enhance their ability to cross the BBB [34]. These carriers can protect the peptides from degradation and can potentially exploit transport mechanisms at the BBB, improving their brain bioavailability.

Therefore, we evaluated the LyeTx III peptide in a solution and encapsulated it in liposomes composed of POPC in HN13 and HaCaT cells. The encapsulation of LyeTx III increased its cytotoxic activity in the tumor cell line. Studies have shown that liposome-encapsulated AMPs exhibit enhanced inhibitory effects against various tumor cells [35], suggesting a potential for these formulations in future anticancer therapies. In addition, previous studies with Melittin, a peptide isolated from bee venom, showed that the blank liposomes exhibited low cytotoxicity, suggesting that the preparation has favorable biological safety and also that its cytotoxic activity was improved in melanoma [36] and lung cancer cell lines. [37]. However, LyeTx III in liposomes also showed a relative toxicity to the control cells, HaCaT, although it was about 20% less that that observed to cancer cells. This point could be improved, for example with some chemical modification of the peptide. One successful example was the pegylated LyeTxI-b, which maintained its antimicrobial activity and significantly diminished its toxicity in mice and its hemolytic activity, compared with the unpegylated peptide [38]. This is the first work showing this new molecule with promising cytotoxic and antiproliferative activity, but further studies are needed to elucidate the mechanisms associated with this activity and also to understand the interaction between the peptide and the cell membranes of control and cancer cells.

## 4. Conclusions

In conclusion, the peptide LyeTx III demonstrated typical characteristics of AMPs and, when encapsulated, showed selective cytotoxic activity against HN13 tumor cells in culture. This result, together with its low similarity with other known antimicrobial peptides in databases, emphasizes the potential of LyeTx III as a novel candidate for anticancer agents.

## 5. Materials and Methods

The spider venom was obtained and processed according to the method described by Santos et al., 2010 [17]. Following the protocol, the collected venom was diluted in Milli-Q water, frozen in liquid nitrogen, and subsequently lyophilized.

### 5.1. Purification of LyeTx III

Cation exchange chromatography and reverse-phase chromatography were conducted according to Santos et al. [17] with some modifications.

#### 5.1.1. Cation Exchange Chromatography

The lyophilized venom was solubilized in Milli-Q water and loaded onto a cation exchange chromatography (CIEX) column (TSK gel CM-SW, 250 mm × 4.6 mm, Tosoh, Japan), adjusted with solution A (10 mM sodium acetate buffer, pH 5). A linear salt gradient was carried out by raising the proportion of solution B (10 mM sodium acetate buffer with 1 M NaCl, pH 5), varying from 0 to 85% of solution B over 18–118 min, 85 to 100% of solution B from 118 to 125 min, and 100% solution of B from 125 to 128 min. The flow rate was set to 0.8 mL·min^−1^ and measured at 214 nm, and the fractions were collected manually [17].

#### 5.1.2. Reverse-Phase Chromatography

The lyophilized fractions acquired from CIEX were dispersed in 0.1% aqueous trifluoroacetic acid (TFA) in Milli Q water and injected onto a reverse-phase chromatography (RPC) column (Supelcosil™ C18, 25 cm × 4.6 mm, Tosoh, Tokyo, Japan), equilibrated with 0.1% aqueous TFA. Elution was performed at a flow rate of 5 mL·min^−1^ with the following solutions: 0–5 min, a gradient of 0–45% acetonitrile in 0.1% TFA in water; 5–40 min, a gradient of 45–60% acetonitrile in 0.1% TFA in water; 40–45 min, gradient of 60–100% acetonitrile in 0.1% TFA in water; 45–55 min, 100% acetonitrile in 0.1% TFA; and 55–60 min, a gradient of 100–0% acetonitrile in 0.1% TFA in water. The flow rate was 1 mL·min^−1^, with detection carried out at 214 nm [17].

### 5.2. Mass Spectrometry

#### 5.2.1. MALDI-TOF-MS

The fractions were analyzed by mass spectrometry performed on a MALDI TOF (AutoFlex III, Bruker Daltonics, Bremen, Germany) in linear and reflector modes, and the spectra were processed with a FlexAnalysis 3.3 (Bruker Daltonics, Germany). Briefly, solubilized fractions (0.5 mL of sample, variable concentrations) were spotted onto the target, followed by 0.5 mL of a CHCA (α-cyano-4-hidroxycinnamic acid) matrix solution (60% acetonitrile/0.3% TFA), and allowed to dry at room temperature (dried-droplet method). Peptide Calibration Standard II (700–4000 Da) and Protein Calibration Standard I (3000–25,000 Da) (Bruker Daltonics, Germany) were used as external calibrates. The mass spectra from the average of 256 laser pulses from *m*/*z* 600 to 22.000 were obtained.

#### 5.2.2. FT-ICR Mass Spectrometer

The sample was analyzed directly using the FT-ICR mass spectrometer, model 9.4 T Solarix (Bruker Daltonics, Bremen, Germany) [39,40,41], equipped with an electrospray ionization (ESI) source operating in negative-ion mode (ESI(-)) and set to cover a mass range of *m*/*z* 150–1500. The mass spectra were externally calibrated using a D-arginine solution within the same mass range (*m*/*z* 150–1500). The ESI source parameters were as follows: nebulizer gas pressure of 1.3 bar, capillary voltage of 3.2 kV, transfer capillary temperature of 200 °C, and skimmer voltage of −30 V. Collision-induced dissociation (CID) [B] experiments were performed with the mass quadrupole resolution, adjusted to isolate 1 *m*/*z* unit, and with the collision energy ranging from 10 to 40 V. The mass spectra were acquired and processed using DataAnalysis 4.0 software (Bruker Daltonics).

#### 5.2.3. Edman Degradation

The fraction containing approximately 3.0 nmols of the peptides was lyophilized, resuspended in 30 μL of 0.1% TFA aqueous solution, and applied directly to a glass fiber membrane (glass fiber disk, TFA-treated, Wako, Japan) to be used in the PPSQ-21A Protein Sequencer (Shimadzu), coupled to the separation of PTH amino acids by reverse-phase chromatography on a WAKOSIL-PTH column (4.6 × 250 mm, 5 μm, Wako, Japan).

### 5.3. Peptide Synthesis and Purification

The peptide was synthesized manually using stepwise solid-phase synthesis based on the N-9-fluorenylmethyloxycarbonyl (Fmoc) strategy (Chan and White, 2000) [42] on a Rink amide resin (0.40 mmol/g) [43]. Side-chain protection groups used included t-butyl for methionine, t-butyloxycarbonyl for lysine, and 2,2,4,6,7-pentamethyldihydrobenzofuran-5-sulfonyl for arginine. Couplings were performed using 1,3-diisopropylcarbodiimide and 1-hydroxybenzotriazole in N,N-dimethylformamide (DMF), with reaction times ranging from 60 to 180 min. Deprotections were achieved by treating the resin twice with piperidine:DMF (1:4, *v*/*v*) for 10 min each. The peptide was separated from the resin and underwent final deprotection using a mixture of TFA, thioanisole, water, 1,2-ethanedithiol, and triisopropylsilane (86.5:5.0:5.0:2.5:1.0, by volume) under standard room conditions for 60 min. The product was precipitated with cold diisopropyl ether, and the crude peptide was extracted using 50% aqueous acetonitrile by volume. The extract was then freeze-dried and purified by reverse-phase high-performance liquid chromatography (RP-HPLC) on a C18 semi-preparative column (Supelco, 5 µm, 250 mm × 10 mm), equilibrated with 0.1% aqueous trifluoroacetic acid (TFA). Elution was performed using a linear gradient of acetonitrile in 0.1% TFA. This started with 0.1% TFA in water, followed by a gradient of 0–20% acetonitrile in 0.1% TFA in water, 20–52% acetonitrile containing 0.1% TFA in water, 52–100% acetonitrile with 0.1% TFA in water, and ending with 100% acetonitrile with 0.1% TFA, within the following times: from 0 to 4 min, from 4 to 14 min, from 14 to 44 min, from 44 to 50 min, and from 50 to 60 min, respectively. The flow rate was set to 3.0 mL/min, with detection carried out at 220 nm.

### 5.4. Preparation of POPC Liposomes and Leakage of Calcein Assay

L-α-phosphatidylcholine was dissolved in chloroform and dried under a nitrogen flow. The lipid film was completely dried under vacuum at 55 °C for 45 min and then hydrated with 1 mL of incubation buffer (0.02 mol·L^−1^ HEPES, 0.15 mol·L^−1^ NaCl, pH 7.2) or 1 mL of 0.02 mol·L^−1^ HEPES buffer, 0.15 mol·L^−1^ NaCl, 0.075 mol·L^−1^ calcein, pH 7.2, for the fluorescence release experiments and measured by spectroscopy. The suspension was sonicated (Sonics–Vibracell) under a nitrogen flow in an ice-water bath until the formation of unilamellar vesicles occurred [40]. The calcein that was not encapsulated in the process was removed by chromatography on a Sephadex G-50 column (1 mL volume), equilibrated with the incubation buffer.

Membrane permeabilization activity was detected by calcein release. The release was monitored in a spectrofluorimeter (Cari Eclipse–Varian), using a wavelength of 490 nm for excitation and 515 for emission. The maximum emitted fluorescence (100% calcein release) was determined by adding 10 μL of Triton X-100 solution (1% by volume) to the sample (final volume: 2.5 mL). Fluorescence was monitored throughout the experiment; the peptide was added after 2 min of incubation, and the temperature was maintained at 37 °C.

### 5.5. Circular Dichroism Spectroscopy

The secondary structure of LyeTx III was analyzed at 25 °C in three different media: a 100 µM phosphate buffer at pH 7.0, a 100 µM Tris–HCl buffer at pH 8.0, and water/TFE mixtures. Measurements were conducted using a Jasco-715 spectropolarimeter (Jasco, Tokyo, Japan) with a 1.0 mm rectangular quartz cuvette (Uvonic Instruments, New York, NY, USA). Spectra were registered from the 260 to 190 nm range, with a spectral bandwidth of 1.0 nm, a step resolution of 0.1 nm, a scan rate of 100 nm/min, and a response time of 4 s. The peptide concentration was maintained at 10 µM for all circular dichroism (CD) investigations, and the resulting spectra were examined using CDPro software (http://lamar.colostate.edu/~sreeram/CDPro).

### 5.6. Cell Culture

A head and neck cancer tumor cell line (HN13) and non-tumor keratinocyte cell line (HaCaT), from clinical surgical samples, were obtained from Hospital do Cancer de Barretos. To perform the experiments, HN13 cells were maintained in a DMEM culture medium (Sigma-Aldrich D5648m St. Louis, MO, USA), supplemented with 10% fetal bovine serum (FBS) (Vitrocell S0011) and 1% streptomycin/penicillin (Gibco 15140-122, Grand Island, NY, USA). The HaCaT cell line was maintained in a DEMEM (SigmaAldrich D5648) and F-12 Ham (Sigma-Aldrich N6760) culture medium (1:1), supplemented with 20% SFB and 1% streptomycin/penicillin. The cells were maintained in an incubator in a 5% humidified atmosphere (CO_2_) at 37 °C.

### 5.7. Cell Viability Assay

To perform the MTT (3-[4,5-dimethylthiazol-2-yl]-2,5-diphenyltetrazolium bromide; M6494, Invitrogen, USA) test, 96-well plates were seeded with 5 × 10^3^ cells per well. They were then incubated overnight in a CO_2_ oven (5%) at 37 °C to achieve adherence on the plate. After 24 h, the treatment was applied. The isolated peptide and liposomes were diluted in a buffer (1%) at concentrations of 45 and 100 µM (triplicate, 24 h), whereas in the control, only the buffer was used. After 24 h of treatment, 100 μL of MTT (0.5 mg·mL^−1^), diluted in a DMEM culture medium, was added to each well, and then, the plate was incubated again for 3 h. Afterwards, the MTT was removed, and 100 μL of DMSO was added for cell lysis and solubilization of formazan crystals, and the absorbance of the samples was measured in a microplate reader at 570 nm [44].

### 5.8. Statistical Analysis

The results obtained were analyzed using GraphPad Prism software version 8.0. The significance level in all statistical analyses was considered significant when the *p*-value ≤ 0.05.

## Figures and Tables

**Table 1:** Sequence alignment of LyeTx III against other amphipathic antimicrobial peptides from spider of *Lycosidae* family.

Peptide	Sequence	MW *	Net Charge	Identify (%)	Ref.
Lyetx III	----G**K**AM**K**AIA**K**FLG**R**----------	1389.76	+5		
Lyetx I	-IWLTAL**K**FLG**K**NLG**K**HLA**K**QQLA**K**L	2831.73	+6	38.46	[17]
LyeTx II	----AGLG**K**IGALIQ**K**VIA**K**Y**K**A---	1940.21	+5	8.33	[18]
LyTx I	--IWLTAL**K**FLG**K**HAA**K**HLA**K**QQLS**K**L	2841.71	+6	23.08	[19]
LyTx II	**K**I**K**WF**K**TM**K**SIA**K**FIA**K**EQM**KK**HLGGE	3204.80	+6	53.85	[19]

The sequences were aligned with Clustal Omega, using a PAM 250 matrix. * MW: Monoisotopic mass.

**Table 2:** Cell viability (%) of the treatments with the LyeTx III peptide alone and liposomes in the HN13 tumor and non-tumor HaCaT.

Cell Viability (%) 24 h		
Treatment	Cell Line	
	HN13	HaCaT
LyeTx III alone	98	74
LyeTx III liposomes	38	56

## Data Availability

The original contributions presented in this study are included in the article. Further inquiries can be directed to the corresponding authors.
